# Clinical nursing interns’ perceptions of artificial intelligence-assisted tools in human–AI collaboration: a qualitative persona-based study

**DOI:** 10.3389/fpubh.2026.1884728

**Published:** 2026-07-02

**Authors:** Liping Yao, Hang Wang, Jianping Liu, Chunmei Song

**Affiliations:** 1 School of Nursing‌, Zhejiang Chinese Medical University, Hangzhou, China; 2 School of Medicine, Shanghai East Hospital, Tongji University, Shanghai, China; 3 Department of Neurology, Yancheng Third People’s Hospital, Affiliated Yancheng Third People’s Hospital of Jiangsu Medical College, Yancheng, China; 4 Department of Neurology, Affiliated Hospital No. 6 of Nantong University, Nantong, China; 5 Department of Disinfection Supply Center, Yancheng Third People’s Hospital, Affiliated Yancheng Third People’s Hospital of Jiangsu Medical College, Yancheng, China; 6 Department of Disinfection Supply Center, Affiliated Hospital No. 6 of Nantong University, Nantong, China

**Keywords:** artificial intelligence, clinical nursing interns, human–AI collaboration, nursing education, qualitative research, user profiles

## Abstract

**Objective:**

This study aims to explore the cognitive characteristics and group differences of clinical nursing interns regarding the use of artificial intelligence-assisted tools in a human–AI collaboration context through a persona-based approach, providing references for optimizing nursing internship teaching and the standardized application of AI-assisted tools.

**Methods:**

A descriptive qualitative research approach was adopted. From January to March 2026, 25 clinical nursing interns were selected through purposive sampling in three tertiary grade A hospitals in Zhejiang and Jiangsu provinces for semi-structured interviews. The interview data were analyzed using Colaizzi’s seven-step method and managed and coded with NVivo 15.0 software. Based on the extraction of cognitive features and label dimensions, a cognitive profile of clinical nursing interns’ use of artificial intelligence-assisted tools was constructed. Following thematic analysis, participants with similar cognitive characteristics, behaviors, and support needs were grouped to construct five distinct persona types.

**Results:**

A total of 25 clinical nursing interns were included. The cognitive profile dimensions of clinical nursing interns regarding artificial intelligence-assisted tools include motivation for use, behavior patterns, trust attitude, degree of dependence, risk awareness, and support needs, etc. Finally, five cognitive profile types were constructed: Active-Thinking type, Process-Adaptation type, Task-Dependent type, Passive-Burden type, and Doubtful-Defense type.

**Conclusion:**

This study is among the first qualitative investigations to apply persona construction to explore nursing interns’ perceptions of AI-assisted tools. In the human–AI collaboration model, clinical nursing interns have significant heterogeneity in their perception of artificial intelligence-assisted tools. Nursing educators should provide personalized teaching guidance, process support, and risk management based on the cognitive characteristics and needs of different types of interns, helping them improve their learning and work efficiency while developing the ability to make independent judgments, use in a standardized manner, and collaborate prudently.

## 1 Background

In recent years, with the rapid development of digital technologies such as artificial intelligence (AI), big data, and the Internet of Things, the human–AI collaboration model has gradually entered the medical field. Humans and intelligent systems form deep collaboration through cognitive consensus and dynamic interaction, effectively enhancing work quality and efficiency (1). In the field of clinical nursing, AI-assisted tools have gradually been applied in areas such as condition monitoring, emergency triage, risk screening, clinical decision-making, health management, and patient follow-up (2). Relevant studies show (3) that the global shortage of nursing staff has reached 6 million. In China, due to the acceleration of population aging and the increase in disease burden, higher requirements have been imposed on the healthcare service system and the quality of nursing. The application of artificial intelligence-assisted tools provides new possibilities for improving the quality of nursing services. Clinical nursing practice is an important stage for nursing students to transition from school education to clinical practice. Its core lies in helping students become familiar with the real clinical environment, complete the role transformation (4), and gradually develop clinical thinking, operational skills and professional identity (5). Nursing interns, as the reserve force of the nursing professional talent pool, their understanding, acceptance and usage experience of artificial intelligence-assisted tools in the human–AI collaborative mode will affect the clinical transformation efficiency of nursing intelligent technologies as well as the digital literacy and professional capabilities of the future nursing workforce. More and more clinical nursing interns are exposed to or using AI-assisted tools in aspects such as vital sign monitoring and virtual simulation training to enhance their work efficiency and reduce the risk of operational errors (6). Although AI-assisted tools have provided many conveniences for clinical nursing interns, they also have certain limitations in their application. For instance, there are differences in the understanding of AI-assisted tools among nursing interns, and prolonged use by them may lead to excessive dependence and a decline in their critical thinking abilities (7). Furthermore, there is also a risk of leakage in terms of the accuracy of AI-assisted tools and the security of private data (8). At present, the research on AI-assisted tools for nursing interns conducted both domestically and internationally mainly focuses on the effectiveness of educational applications (9), their attitudes towards AI-assisted tools, as well as ethical and risk aspects. There is a lack of in-depth studies on the experiences and influencing factors of clinical nursing interns when using AI-assisted tools in a human–AI collaborative mode. Although previous studies have examined attitudes toward AI among nursing students (10), little is known about the heterogeneity of nursing interns’ experiences and cognitive patterns when interacting with AI-assisted tools in clinical settings. Most prior study has also examined this one theoretical lens at a time. Some studies apply technology adoption models such as the Technology Acceptance Model (TAM), asking how perceived usefulness and ease of use shape the intention to use AI (11). Others draw on digital and AI literacy frameworks, including DigComp 3.0, which set out the competencies needed to understand, evaluate, and use AI tools appropriately (12, 13). A third line of research treats trust in AI as multidimensional, shaped by reliability, transparency, and explainability (14, 15). What remains unclear is how these factors come together in any one intern. User profiling technology is a virtual image based on data mining and real user data. It can categorize individuals with common characteristics and needs and form a typical group image. By presenting the characteristics of different subgroups, the heterogeneity within the target population is explained, and character roles are formed. Personas, as described by Pruitt and Grudin, refer to representative descriptions formed by synthesizing the heterogeneity within a user group (16). They are research-based profiles of users who share common characteristics, behaviors, needs, and goals. We selected a persona-based approach because it captures the heterogeneity among nursing interns and translates their differing perceptions and support needs into targeted educational and clinical guidance. Therefore, this study adopted a qualitative research method, using semi-structured interviews to construct user profiles. The aim was to explore the differences in the cognition, experience and demands of clinical nursing interns regarding AI-assisted tools in the context of Chinese culture, under the human–AI collaboration context. Based on the five user profile types derived from the interview data, this study may provide a practical framework for designing differentiated AI-use training, clarifying the boundaries of AI application, and optimizing AI-assisted tools from a user-centered perspective. This study aims to provide scientific evidence for promoting the development of AI-assisted tools and nursing internship teaching and cultivating future nursing professionals with digital literacy.

## 2 Methods

### 2.1 Research design and participants

This study adopted a descriptive qualitative research design. A descriptive qualitative design was selected because the study aimed to obtain a comprehensive description of interns’ perceptions and experiences rather than develop a formal theory or explore lived experiences at a phenomenological level. Through in-depth interviews, it explored the perceptions and demands of clinical nursing interns regarding the use of artificial intelligence-assisted tools. The research was conducted from January to March 2026 in three tertiary grade A hospitals in Zhejiang and Jiangsu. To ensure the depth of the research results, we employed purposive sampling to select participants from clinical nursing interns who used artificial intelligence-assisted tools. Participants were purposively selected to ensure variation in gender, educational level, internship duration, department, and frequency of AI tool use. The sample size was determined based on the data saturation principle, which means the interviews should be terminated when no new information emerged in the third consecutive session. The research process strictly followed the Consolidated Criteria for Reporting Qualitative Research (COREQ) guidelines for qualitative research reports (17) to ensure the rigor, transparency, and reproducibility of the research process.

#### 2.1.1 Inclusion and exclusion criteria

Inclusion criteria: (1) completed the basic theory courses of nursing and had clinical internship for at least 1 month; (2) during the internship, there was direct or indirect experience of using or coming into contact with such tools; (3) possessing clear language expression ability, able to accurately describe one’s own feelings about using or coming into contact with artificial intelligence-assisted nursing tools; (4) informed consent, able to participate in the interview.

Exclusion criteria: (1) interns who are not from the nursing major, with an internship duration of less than 1 month, and who have not completed the basic theoretical learning of the nursing major; (2) interns whose clinical department did not apply artificial intelligence-assisted nursing tools during the internship, and who never came into contact with or used such tools during the internship process; (3) those who cannot complete the interview due to physical, psychological or language communication reasons; (4) those who withdrew from the study or had severely missing interview materials during the process; (5) those who have communication barriers or cannot cooperate with the interview.

### 2.2 Data collection and organization

This study employed a qualitative research approach, collecting data through semi-structured in-depth interviews between pairs. The research team established cooperation with the clinical practice supervisors of three hospitals in Zhejiang Province and Jiangsu Province. They clearly explained the purpose, significance and process of this research, and specified that the clinical practice supervisors of each hospital would act as recruitment assistants, responsible for the initial screening and communication of clinical interns meeting the inclusion criteria within the hospital. The list of potential participants identified through the initial screening was provided to the research team. Researchers who had received training in qualitative research drafted standardized recruitment information sheets, and the clinical practice supervisors issued the recruitment announcement through an intern symposium. If potential participants are willing to participate, they can contact the researchers themselves. The researchers will conduct a second qualification check on them, confirming one by one whether they fully meet the inclusion and exclusion criteria. Those who pass the check will be listed as potential interview candidates. Ultimately, this study recruited a total of 32 clinical nursing interns who met the inclusion criteria. However, 4 of them were unable to participate in the interviews due to scheduling conflicts. Before the formal interviews, 3 of them were given a pre-interview. Since the purpose of the pre-interview was to test the scientific and feasibility of the interview outline, the interview contents were not included in the research results. Formal interviews and data analysis were conducted concurrently. Data saturation was monitored through continuous comparison of codes and themes during data collection and analysis. When the interviews reached the 23rd participant, the main codes, themes, and persona characteristics had become relatively stable, with no new important information emerging. No new codes emerged in the final three interviews. To further confirm data saturation, the 24th and 25th participants were interviewed, and no new themes or persona characteristics were identified, indicating that data saturation had been achieved. Eventually, 25 clinical nursing interns were included in the study. Before the formal interviews, general sociodemographic information was collected, including age, gender, educational level, current internship department, duration of internship, frequency of using AI-assisted tools, previous AI-related training experience, prior use of ChatGPT or other generative AI tools, self-reported digital literacy, and types of AI-assisted tools. This information is crucial for analyzing the relationship between participants’ cognition of using AI-assisted tools and their social background. Data was collected through face-to-face in-depth interviews, conducted in quiet meeting rooms or online video meeting rooms. Each interview was conducted by two main researchers and lasted for 30–60 min. The interviews were conducted by two researchers with nursing academic backgrounds and formal training in qualitative interviewing. Both researchers had experience in qualitative data collection and had received standardized training on the interview guide before the formal interviews. The interviewers had no prior personal or supervisory relationship with the participants. With the participants’ consent, the interview process was recorded, and the recordings were transcribed within 24 h.

#### 2.2.1 Interview guide

Based on the research objectives, a semi-structured interview guide was developed to deeply explore the participants’ cognitive situation regarding the use of artificial intelligence-assisted tools. During the compilation process, we invited 6 experts to participate in the consultation. Among them, 2 were head nurses, 2 were departmental teaching instructors, 1 was a qualitative research expert, and 1 was a professor from the nursing college. The research team sent the preliminary interview guide to 6 experts via the internet to inquire whether the interview outline was comprehensive, reasonable, and reliable. The 6 experts independently proposed revision suggestions, and the research team summarized the experts’ opinions and held an online group meeting to discuss and revise the existing issues. The revised interview guide was then sent to the 6 experts for verification again. None of the experts raised new opinions. Subsequently, pre-interviews were conducted with 3 recruited participants and the guide was revised based on their feedback. Following the pilot interviews, several questions were revised to improve clarity and facilitate deeper exploration of interns’ AI use experiences. The core questions of the final interview guide are shown in Table 1.

**Table 1:** Core questions of the interview guide.

Theme	Questions
Basic cognition	1.What AI-assisted nursing tools have you encountered during your clinical internship?
	2.Through what channels did you learn about or access these AI-assisted nursing tools? How did you feel when you first encountered them?
User experience	3.How frequently do you use these AI-assisted tools during your internship? In what scenarios have you used them? Was the experience smooth? What difficulties have you encountered?
	4.What benefits do you think these AI-assisted tools have brought to your internship work? Have they had any negative effects?
	5.Are you willing to continue using AI-assisted tools in your future nursing work? Are you willing to actively learn about new AI-assisted tools?
	6.Have you had any confusion or concerns when using AI-assisted tools?
Needs and expectations	7.What support do you hope schools or internship hospitals will provide regarding guidance on the use of AI-assisted tools?
	8.What suggestions do you have for improving the AI-assisted nursing tools currently used in clinical practice?
	9. What features do you wish AI-assisted tools had but currently do not provide?
Others	10.Is there anything else you would like to add?

### 2.3 Data analysis

#### 2.3.1 Establishment of the dimension of cognitive portrait labels

Portrait construction typically involves three steps: data collection, feature extraction, and portrait presentation. After completing the collection of semi-structured interview data in this study, we used the Colaizzi seven-step analysis method (18) to analyze the interview texts and utilized NVivo 15.0 software for data organization and coding. The analysis process includes familiarizing with the data, extracting meaningful statements, forming codes, summarizing themes, providing detailed descriptions, establishing a basic structure, and verifying the results. The analysis processing involves two researchers repeatedly reading the interview data, independently extracting statements related to the use of artificial intelligence-assisted tools and forming corresponding codes. Then, these codes are reviewed to determine the types and themes, and they are combined to form the common characteristics of the participants. These common characteristics constitute the basic dimensions of the personality labels for nursing interns, used to describe the behaviors and cognition of the participants. When the research results of the two researchers differ, the research team discusses and reaches a consensus.

#### 2.3.2 Extraction of cognitive portrait features and portrait construction

**Figure 1:**
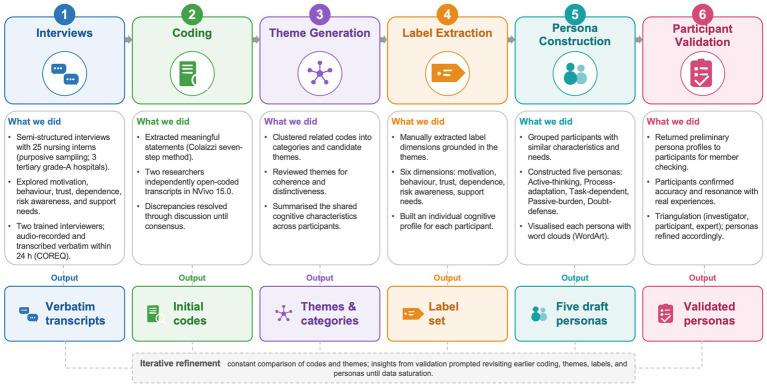
Persona development process.

Based on the dimensions of the cognitive profile labels, the researchers combined the interview texts and the results of thematic analysis and adopted a manual extraction method to analyze the characteristics of the participants in each dimension. The research team integrated the characteristics of each participant through discussion to form personalized cognitive profiles. Then, based on the characteristics of each dimension of the participants, they analyzed and grouped those with similar characteristics and needs together. Finally, they summarized different types of group profiles. Subsequently, they fed back the preliminary profile results to the participants for verification, confirming whether the profile features matched their real experiences and thoughts, and making revisions based on the feedback. After discussion by the research team, it was believed that the constructed profiles could well reflect the characteristics of the participants. The overall process of persona development is shown in Figure 1.

#### 2.3.3 Criteria for persona classification

Classification was based on the six cognitive-label dimensions established earlier: motivation for use, behavior patterns, trust attitude, degree of dependence, risk awareness, and support needs. The features extracted for each participant across these dimensions formed that participant’s cognitive profile, and the profile as a whole, not any single dimension, formed the basis for classification. Grouping followed two rules. Participants whose profiles showed similar dimensional patterns and comparable support needs were placed in the same type. When a profile carried features of more than one emerging group, assignment was decided by the pattern the participant expressed most strongly and most consistently across the interview. The types thus emerged from this grouping and were not defined in advance. Two researchers carried out the process independently. Each read the participant’s transcript, codes, and extracted dimensional features, then assigned a preliminary type. Where their judgments differed, the case was re-examined against the original interview data and representative quotations and taken to the research team for discussion until consensus was reached. The resulting persona types and their characteristics are reported in the Results.

#### 2.3.4 Presentation of cognitive profiles

Portrayal presentation is the process of expressing groups with different characteristics in a visual form. Common forms of portrayal presentation include word clouds, character images, statistical charts, and labeled descriptions, etc. This study used the Word Art software to draw word clouds, visually presenting the characteristic portraits of clinical nursing interns’ cognition of artificial intelligence-assisted tools. Through visual portrayal, the diversity of cognition of clinical nursing interns in using artificial intelligence-assisted tools in the human–AI collaborative mode was presented.

### 2.4 Rigor

This study adheres to the guidelines for qualitative research reports. Both the data collectors and the analysts have received training in qualitative research and possess the ability to conduct interviews and data analysis. Before the interviews, the researchers explained the purpose, significance, use of the data, and confidentiality principles to the participants, reasonably arranged the interview time and environment to establish a trust relationship. During the interviews, the researchers maintained a neutral attitude and avoided using suggestive or leading language. Researchers maintained reflexive journals throughout data collection and analysis to identify and manage potential biases. The interviews were conducted jointly by two researchers, one conducting a formal face-to-face interview and the other responsible for recording non-verbal information. After the interviews, the audio recordings, transcribed texts, and interview notes were promptly organized. The data analysis was carried out separately by two researchers for coding and theme extraction, and consensus was reached through discussion. The study adopted the triangulation verification method, including researcher analysis, participant verification, and expert evaluation. Triangulation in this study took three forms. In the first, investigator triangulation, two researchers coded the transcripts independently, then compared their results and resolved disagreements through discussion with the wider research team. Participant validation came next. Participants reviewed summaries of the preliminary persona profiles and key findings, and confirmed the accuracy of the interpretations. The third was expert review, in which specialists in qualitative research and nursing education examined the coding, theme generation, and persona classification and judged their credibility and appropriateness. The research team fully preserved the interview outline, transcribed materials, coding process, and analysis records to ensure the traceability of the research process. These rigor strategies were mapped to the COREQ domains of researcher reflexivity, study design, data analysis, and reporting of findings to enhance methodological transparency.

### 2.5 Ethics statement

This study was approved by the Ethics Committee of Yancheng Third People’s Hospital (2025-128). All participants signed an informed consent form before the interview. Throughout the entire research process, the researchers strictly protected the privacy and personal information of the participants. All audio recordings and transcribed texts were used solely for the analysis of this study. Without the consent of the participants, they cannot be disclosed or used for any other purposes. When reporting the results, the participant information was anonymized to ensure full protection of their privacy.

## 3 Results

### 3.1 The demographic characteristics of the participants

A total of 25 clinical nursing interns were included in this study, as shown in Table 2. Among them, 9 were male and 16 were female. Their ages ranged from 20 to 23 years old, with an average age of 21.8 years. There were 17 undergraduate students, accounting for 68.00%, and 8 junior college students, accounting for 32.00%. The internship departments covered internal medicine, surgery, emergency department, ICU, pediatrics and gynecology, pediatric and geriatric, rehabilitation, and oncology-related departments, among which the internal medicine-related departments accounted for the highest 28.00%. The duration of internship was mostly more than 6 months, accounting for 76.00%. The frequency of using artificial intelligence-assisted tools was mainly 3–5 times per week. Regarding AI-related experience, most participants had prior experience using ChatGPT or other generative AI tools, while relatively few had received formal AI-related training. Their self-reported digital literacy was mainly at a moderate level. The types of usage were mainly large language model question-answering tools, mobile nursing information systems, and intelligent monitoring or warning devices.

**Table 2:** General population data.

Item	Category	Number/mentions (*n*)	Percentage (%)
Age/years	20	2	8.00
	21	7	28.00
	22	10	40.00
	23	6	24.00
Gender	Male	9	36.00
	Female	16	64.00
Educational level of institution	Junior college	8	32.00
	University	17	68.00
Current internship department	Internal medicine-related departments	7	28.00
	Surgery-related departments	6	24.00
	Emergency department/ICU	5	20.00
	Obstetrics, gynecology, and pediatrics	3	12.00
	Geriatrics/rehabilitation/oncology-related departments	4	16.00
Internship duration	<3 months	2	8.00
	3–6 months	4	16.00
	>6 months	19	76.00
Frequency of using AI-assisted tools	<3 times per week	5	20.00
	3–5 times per week	12	48.00
	>5 times per week	8	32.00
Previous AI-related training	Formal course or lecture	3	12.00
	Clinical demonstration or departmental guidance	5	20.00
	Informal self-learning or online exposure	6	24.00
	No specific training	11	44.00
Prior use of ChatGPT or other generative AI tools	Frequently used for learning or documentation	7	28.00
	Occasionally used for information searching or Q&A	11	44.00
	Rarely used	4	16.00
	No prior use	3	12.00
Self-reported digital literacy	Able to use AI-assisted tools independently	6	24.00
	Able to use common digital tools with guidance	14	56.00
	Limited confidence in using digital or AI-assisted tools	5	20.00
Types of AI-assisted tools	Large language model-based Q&A tools	18	23.38
	Mobile nursing information systems	17	22.08
	Intelligent monitoring or early-warning devices	14	18.18
	Virtual simulation training systems	10	12.99
	Intelligent auxiliary functions in hospital information systems	9	11.69
	Intelligent nursing assessment systems	5	6.49
	Intelligent health education tools	4	5.19

The types of AI-assisted tools were classified from participants’ own accounts of what they had encountered or used during clinical internships. Large language model-based Q&A tools are generative AI tools used to answer questions, explain disease-related knowledge, organize nursing problems, or draft health education content. Mobile nursing information systems are electronic platforms used at the bedside to check information, complete nursing records, scan barcodes, and log workflow. Intelligent monitoring or early-warning devices automatically gather patient data and alert staff to abnormal vital signs or clinical risks. Virtual simulation training systems are AI-supported platforms for clinical skills training, case-based learning, and scenario-based decision-making. Intelligent auxiliary functions in hospital information systems are built-in features that help with order checking, risk screening, documentation reminders, or information retrieval. Intelligent nursing assessment systems are tools that support the standardized assessment of patient risks or nursing problems, while intelligent health education tools deliver individualized health education, patient guidance, or follow-up information. Previous AI-related training covered anything from formal courses, lectures, clinical demonstrations, and departmental guidance to informal self-learning or incidental online exposure. Prior use of ChatGPT or other generative AI meant participants had drawn on such tools for learning, information searching, question answering, documentation support, or clinical knowledge. Self-reported digital literacy reflected how participants described their own confidence and ability in using digital platforms, mobile nursing systems, and AI-assisted tools.

### 3.2 The character roles of clinical nursing interns using artificial intelligence-assisted tools in the human–AI collaboration mode

This study conducted interviews with clinical nursing interns and ultimately constructed a cognitive profile of clinical nursing interns regarding the use of AI-assisted nursing tools, namely active-thinking type, process-adaptation type, task-dependent type, passive-burden type, and doubtful-defense type. Different roles reflect the cognitive differences, behavioral characteristics and priority needs of nursing interns when using AI-assisted tools during their clinical learning and practice, as shown in Tables 3, 4.

**Table 3:** Demographic characteristics of the five persona types.

Item	Active-thinking type	Process-adaptation type	Task-dependent type	Passive-burden type	Doubtful-defense type
Personas	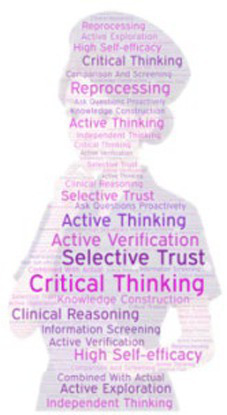	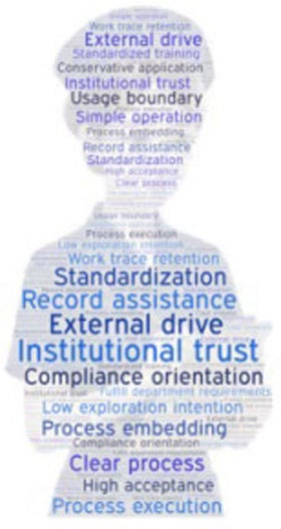	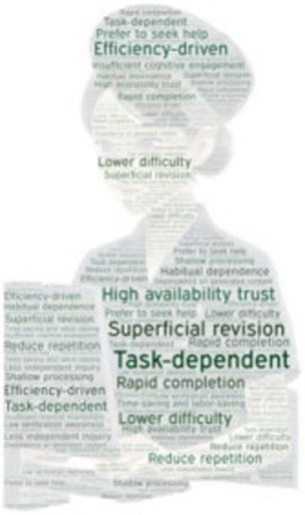	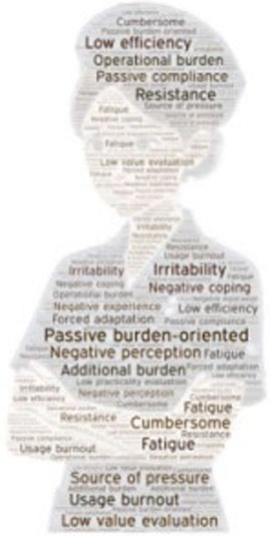	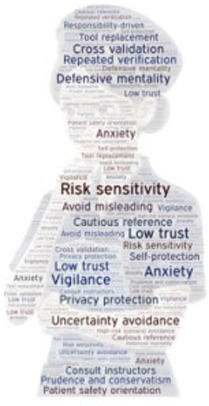
Representative participants	Interns 03, 08, 12, 17, 20	Interns 01, 04, 10, 11, 18, 23	Interns 05, 07, 14, 15, 19, 22	Interns 02, 09, 16, 21, 25	Interns 06, 13, 24
Gender, n	Male: 1; female:4	Male: 2; female: 4	Male: 2; female: 4	Male: 2; female: 3	Male: 2; female: 1
Educational level, n	Junior college student: 1 Undergraduate student: 4	Junior college student: 2 Undergraduate student: 4	Junior college student: 2 Undergraduate student: 4	Junior college student: 2 Undergraduate student: 3	Junior college student: 1 Undergraduate student: 2
Internship duration, n	3–6 months: 1 >6 months: 4	<3 months: 1 3–6 months: 1 >6 months: 4	3–6 months: 1 >6 months: 5	<3 months: 1 3–6 months: 1 >6 months: 3	>6 months: 3
Frequency of use, n	<3 times/week: 1 3–5 times/week: 2 >5 times/week: 2	<3 times/week: 1 3–5 times/week: 4 >5 times/week: 1	3–5 times/week: 2 >5 times/week: 4	<3 times/week: 2 3–5 times/week: 3	<3 times/week: 1 3–5 times/week: 1 >5 times/week: 1
Previous AI-related training experience, n	Formal: 1 Clinical guidance: 1 Informal exposure: 2 None: 1	Formal: 1 Clinical guidance: 2 Informal exposure: 1 None: 2	Formal: 1 Clinical guidance: 1 Informal exposure: 2 None: 2	Clinical guidance: 1 Informal exposure: 1 None: 3	None: 3
Prior use of ChatGPT or other generative AI tools, n	Frequent: 2 Occasional: 2 Rare: 1	Frequent: 1 Occasional: 4 Rare: 1	Frequent: 3 Occasional: 2 Rare: 1	Frequent: 1 Occasional: 1 Rare: 1 None: 2	Occasional: 2 None: 1
Self-reported digital literacy, n	Independent: 3 With guidance: 2	Independent: 1 With guidance: 5	Independent: 1 With guidance: 4 Limited confidence: 1	With guidance: 2 Limited confidence: 3	Independent: 1 With guidance: 1 Limited confidence: 1

**Table 4:** Persona characteristics of clinical nursing interns’ perceptions of AI-assisted tools.

Item	Active-thinking type	Process-adaptation type	Task-dependent type	Passive-burden type	Doubtful-defense type
Cognitive dimension of AI-assisted tools	Learning support and thinking-expansion tools	Clinical workflow and documentation assistance tools	Task completion and efficiency-improvement tools	Sources of operational burden and stress	Cautious reference tools with risk uncertainty
Motivation for use	① Knowledge supplementation ② Thinking expansion ③ Clinical judgment improvement	① Workflow compliance ② Standardized documentation ③ Work traceability	① Task completion ② Time saving ③ Writing burden reduction	① Required system operation ② System compliance ③ Passive use	① Auxiliary reference ② Accuracy concern ③ Safety concern
Behavior patterns	① Active inquiry; ② Information comparison ③ Patient-context integration ④ Instructor consultation	① System-prompt following ② Workflow-based operation ③ Routine recording and verification	① Framework adoption ② Template use ③ Limited revision	① Passive operation ② Repeated checking ③ Resistance to complex procedures	① Repeated verification ② Teacher confirmation ③ Cross-source checking ④ Avoidance in high-risk tasks
Trust attitude	① Selective trust ② Critical acceptance	① Procedural trust ② Trust in hospital-based systems	① Functional trust ② Insufficient critical appraisal	① Low perceived usefulness ② Low acceptance	① Cautious trust ② Uncertainty about clinical reliability
Degree of dependence	① Low to moderate dependence ② Thinking-oriented use	① Moderate dependence ② Procedure- based reliance	① High dependence ② Task-pressure triggered reliance	① Passive dependence ② Low willingness to use	① Low dependence ② Reference-only use
Risk awareness	① Patient-context mismatch ② Judgment support limits	① Data entry errors ② System lag ③ Workflow complexity	① Direct adoption ② Superficial processing ③ Insufficient verification	① Ineffective alerts ② Data desynchronization ③ Workflow disruption	① Information inaccuracy ② Judgment bias ③ Privacy leakage ④ Misleading decision
Support needs	① Structured clinical reasoning training ② Case-based AI appraisal ③ Tool-based information comparison	① Operational routines ② Checklist guidance ③ Clear use boundaries in workflows	① Task-oriented use rules ② Patient-specific evidence supplementation ③ Content verification before adoption	① Workflows introduction redesign ② Graded use requirements ③ Early-stage technical support	① Applicable boundary clarification ② Verification pathways ③ Responsibility attribution

**Figure 2:**
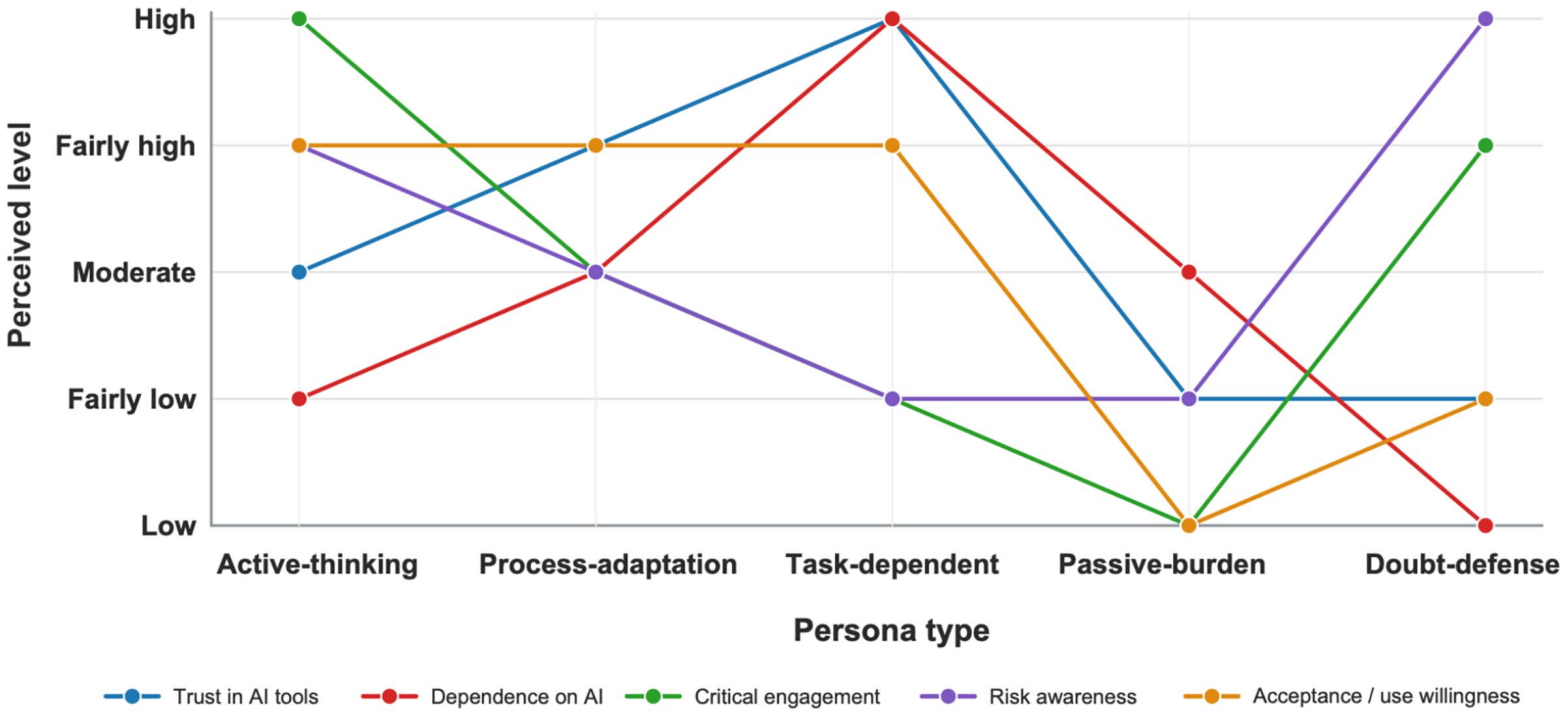
Cognitive profiles of the five personas across key dimensions.

To further illustrate how the five persona types relate to one another, we developed a conceptual figure mapping their relative tendencies across five key dimensions: trust in AI tools, dependence on AI, critical engagement, risk awareness, and acceptance or willingness to use (Figure 2). These levels are qualitative tendencies drawn from the thematic analysis, not measured scores. The personas separate clearly in Figure 2. Active-thinking interns scored high on critical engagement and acceptance while sitting only moderate on trust and dependence. Trust and acceptance were both moderate-to-high among process-adaptation interns, in line with their workflow-driven use of the tools. Task-dependent interns stood out for high dependence paired with weak critical engagement, consistent with their habit of leaning on AI output to get tasks done. Acceptance and willingness to use were lowest in the passive-burden group, who experienced the tools as an added operational load. Doubtful-defense interns sat at the opposite end: high risk awareness, low dependence, and a cautious, verification-first style of use.

#### 3.2.1 Perceptions of AI-assisted tools among active-thinking interns

The active-thinking Interns are more active in using AI-assisted tools. They mainly focus on the roles of AI in knowledge supplementation, idea expansion, and clinical understanding. They regard AI-assisted tools as auxiliary resources for clinical learning and thinking expansion, and actively apply tool-based information to clinical issues. Such interns pay more attention to the value of AI-assisted tools in promoting learning, improving judgment, and enhancing clinical thinking. They usually use AI-assisted tools to understand disease mechanisms, medication effects, nursing problems, and health education content. At the same time, they do not directly equate the output of the AI-assisted tools with clinical conclusions, but rather through comparison, screening, and reprocessing, transform tool-based information into their own knowledge and judgment abilities.

##### 3.2.1.1 Characteristic

- Actively integrates assessment results, monitoring data, nursing records, and risk alerts from clinical AI-assisted nursing tools to further understand patients’ conditions, nursing problems, and nursing interventions.
- The motivation for use is to supplement knowledge, expand thinking, understand clinical logic, and improve judgment ability.
- Integrates content generated by AI-assisted tools with patients’ actual conditions, textbook knowledge, and teachers’ opinions.
- Has selective trust in AI-assisted tools, neither blindly copying their outputs nor completely rejecting them.
- Is able to transform information provided by AI-assisted tools into personal nursing ideas and judgment basis.

##### 3.2.1.2 Demand

The demands of active-thinking interns mainly focus on advanced clinical reasoning support and AI information appraisal training. Case-based guidance can be provided to help them compare AI-generated content with patients’ actual conditions, nursing standards, and instructors’ feedback. In addition, these interns need training in prompt design, evidence verification, and the transformation of AI outputs into individualized nursing judgments, so that AI-assisted tools can be used as resources for expanding thinking.

S03: “It helps me provide a comprehensive perspective, because it’s difficult for one person to have a comprehensive understanding of patient care procedures.”

S08: “When an AI-assisted tool reminds me of something or gives an assessment result, I do not just go by the system prompt. I usually compare it with the patient’s real condition and what my teacher told me.”

S12: “The AI-assisted tools will enhance the critical thinking of nursing students, allowing us to question, understand, and accept. This process is continuous.”

S20: “Our main goal is to continuously enhance our own capabilities, rather than improving the AI’s problem-solving ability. Ultimately, it still needs to be focused on our own ability growth.”

S17: “Sometimes AI points me in a new direction. It might bring up a nursing problem or a health education point I had not thought of. But I still have to decide whether it really suits this patient.”

#### 3.2.2 Perceptions of AI-assisted tools among process-adaptation interns

Process-adaptation interns are those who are exposed to AI-assisted tools mainly through existing hospital systems and routine departmental workflows at an early stage of clinical internship. Their use of these tools is mainly concentrated in basic nursing tasks and work-traceability processes. These interns naturally encounter and use AI-assisted tools in the clinical process, and have a high acceptance of such tools. Their usage behavior is more process-driven rather than exploratory. They usually complete operations such as scanning, inputting, checking, verifying, and recording according to system prompts, department requirements, or demonstrations by the instructors. They have relatively high trust in the existing hospital systems, but have a weak willingness to actively expand the deep functions of AI-assisted tools.

##### 3.2.2.1 Characteristic

- AI-assisted tools have been embedded in nursing workflows and have become part of nursing practice.
- The main purposes of use are to meet departmental requirements, carry out medical orders, complete nursing records, and leave work traces.
- Their behavioral pattern mainly involves operating according to system prompts, departmental workflows, and teachers’ demonstrations.
- They relatively trust hospital-based systems, but have limited willingness to actively explore them.

##### 3.2.2.2 Demand

The demands of process-adaptation interns mainly focus on clear operational guidance and workflow-integrated support. They need standardized training to understand when, where, and how to use AI-assisted tools in routine nursing workflows. Internship departments can provide short operation manuals, demonstration videos, or checklist-based guidance to help interns complete system operations accurately. They also need clear instructions on the boundaries of AI use to reduce uncertainty and improve the accuracy and standardization of clinical workflow adaptation.

S01: “When I first came to the department, I mostly learned the systems by watching how the teacher did it. After that I just followed the fixed steps, scanning, checking, recording, and submitting whatever the system told me to.”

S04: “I think these tools are already part of the nursing work. After each teacher completes the patient care, they need to record what they have done in the system. This is not only a nursing record, but also a work trace.”

S11: “These tools in the department are basically mandatory to use. They cannot be used if one wants to avoid it. Instead, they have been integrated into the daily nursing process.”

S18: “These systems are required to be used throughout the hospital. Therefore, during the internship, we also need to operate according to the requirements of the hospital and the department.”

S23: “For routine work, the system prompts really help, because they remind us what to check or record. When the steps are clear, it’s much easier for us interns to fit into the department’s workflow.”

#### 3.2.3 Perceptions of AI-assisted tools among task-dependent interns

Task-dependent interns use AI-assisted tools more frequently and with clear purposes. Their focus is on handling nursing documents, integrating case data, and completing clinical nursing learning tasks. These interns are mainly oriented towards task completion and have a strong reliance on the functions of AI-assisted tools such as content generation, framework organization, and standardized expression. Their use of AI-assisted tools is primarily efficiency-oriented, aiming to reduce repetitive work and quickly obtain usable frameworks, drafts, or standardized expressions. At the same time, when they encounter tasks, they are accustomed to relying on AI assistance to obtain frameworks, drafts, or templates first, and using the AI output as an important basis for content organization and task completion. When clinical tasks are heavy, time is limited, or knowledge reserves are insufficient, this pattern may further increase their dependence on AI-assisted tools and reduce their initiative in independent analysis and patient-specific verification.

##### 3.2.3.1 Characteristic

- Mainly uses AI-assisted tools for tasks such as nursing documentation, case data organization, nursing care plan development, and completion of internship assignments.
- The main purposes of use are to complete tasks quickly, save time, reduce repetitive writing, and lower task difficulty.
- Often directly enters task requirements and relies on AI to generate frameworks, drafts, templates, or standardized expressions, followed by simple revisions.
- When faced with heavy tasks, time pressure, or insufficient knowledge, they are likely to reduce the process of independently consulting materials, analyzing patient conditions, and organizing nursing ideas.
- They insufficiently verify the accuracy, clinical applicability, and patient-specific relevance of AI outputs, which may lead to risks of direct copying, mechanical application, and superficial processing.

##### 3.2.3.2 Demand

The demands of task-dependent interns mainly focus on preventing overdependence, improving verification ability, and cultivating active thinking. They need explicit rules for AI use, requiring them to verify key information and encouraging them to reflect before adopting AI outputs. For example, when using AI-assisted tools to support nursing work, interns should be guided to verify whether the content matches the patient’s diagnosis, symptoms, treatment plan, and individualized nursing needs. This can help them shift from passively accepting AI outputs to actively analyzing, making critical judgments, and improving clinical reasoning ability.

S05: “When I have to summarize a case or write a nursing plan, I usually let the AI-assisted tool draft a basic structure first. Then I just fill in the patient’s information, so I’m not starting from scratch.”

S07: “When organizing patient information, writing nursing records, or formulating nursing plans, I am accustomed to using AI-assisted tools to generate frameworks and drafts first, and then making simple modifications based on the patient’s condition. This way, the task is completed faster.”

S14: “When time is tight, I’ll just use the framework or wording the AI-assisted tool gives me. I might glance over it for obvious mistakes, but I do not always have time to check every detail.”

S15: “When there are many tasks, my first reaction is to use AI-assisted tools to handle patient information and nursing documents because it can first organize the content, reducing a lot of writing pressure.”

S22: “Nursing records and shift reports need to be written every day. If the AI-assisted tool can automatically generate templates, I will be more dependent on it to organize patient information, and I will mainly be responsible for checking and supplementing.”

#### 3.2.4 Perceptions of AI-assisted tools among passive-burden interns

Passive-burden type interns have more experience with AI-assisted care tools, but they have a lower evaluation of their usage experience. They often feel additional pressure in aspects such as nursing document recording, information entry and verification, system reminders and warnings. For this type of interns, the AI-assisted tools are not perceived as a burden reduction support during their use; instead, they are experienced as additional operational demands in process execution, system operation, and task completion. The burden felt by these interns mainly stems from the mismatch between the system process and actual clinical workflow needs. When using AI-assisted care tools, they often need to repeatedly complete operations such as entry, selection, copying, submission, and verification; when tool design fails to align with nursing workflow, problems such as complex interfaces, cumbersome steps, frequent reminders, slow system response, or data inconsistency may further increase time costs and operational pressure.

##### 3.2.4.1 Characteristic

- Has a relatively negative experience with AI-assisted nursing tools and often feels dissatisfied due to system lag, complex interfaces, frequent reminders, data desynchronization, and rework caused by errors.
- Believes that some AI-assisted tools do not significantly reduce the nursing workload; instead, they increase the time costs of documentation, checking, confirmation, and correction.
- Easily perceives pressure from complicated workflows and task accumulation during nursing documentation, information entry and verification, system reminders, and early-warning processes.
- Has low initiative in use, shows limited willingness to learn and explore new functions, and tends to focus only on completing necessary operations.
- Develops irritability, fatigue, or resistance due to learning costs, repetitive workflows, and operational burdens.

##### 3.2.4.2 Demand

The demands of passive-burden interns mainly focus on reducing operational burden, improving system practicality, and enhancing the fit with clinical workflows. The fit between AI-assisted tools and clinical nursing workflows should be improved by optimizing data synchronization, managing system reminders hierarchically, and simplifying commonly used modules, thereby reducing the additional burden caused by repeated data entry, invalid alerts, and complex operations. In addition, during the early stage of internship, clinical instructors can provide bedside demonstrations, step-by-step guidance, and timely technical support to help interns reduce learning costs and anxiety about use.

S02: “Data between different pages cannot be automatically synchronized. The same information needs to be filled out several times. Once an error is made, it has to be corrected again. Originally, we wanted to improve efficiency, but it turned into a new burden.”

S09: “Nursing records, each patient needs to be written, and the records in some departments are very complicated, just for the sake of writing.”

S16: “Some systems have so many steps, and the screens aren’t easy to follow. As interns, we have to keep both the clinical workflow and the system steps in our heads, so sometimes the tool just feels like one more thing to finish.”

S21: “The system alerts are too frequent. Sometimes, pop-up windows and alarms keep appearing, and the truly important information is easily overlooked. After using it for a long time, one will find it very annoying.”

S25: “When the department is busy, I just want to get the necessary steps done as fast as I can. If the AI-assisted tool keeps reminding me or asking me to confirm things over and over, it only makes me more anxious.”

#### 3.2.5 Perceptions of AI-assisted tools among doubtful-defense interns

The doubtful-defense type of interns have a relatively lower level of trust in AI-assisted care tools and are particularly concerned about potential issues such as content errors, incomplete information, judgment biases, or privacy leaks. Their use of AI-assisted tools is characterized by strong risk awareness and cautious verification. During the usage process, they pay more attention to the accuracy, safety, and applicable boundaries of tool-based information, always maintaining a skeptical and defensive attitude. They often exhibit behaviors such as repeated verification, asking teachers for advice, cross-checking with other sources, or avoiding using AI-assisted tools in complex tasks and high-risk scenarios.

##### 3.2.5.1 Characteristic

- Is doubtful about the content generated by AI-assisted tools, especially showing incomplete trust in generative AI.
- On the one hand, hopes to receive support from AI-assisted tools; on the other hand, worries that these tools may be unreliable.
- Often confirms with teachers after use, or checks again by consulting materials or switching to other tools.
- Is more cautious when using AI-assisted tools for high-risk tasks such as nursing diagnosis, critical value management, and individualized patient assessment.
- Pays close attention to errors, fabrication, data falsification, privacy leakage, and misleading decision-making caused by AI-assisted tools.

##### 3.2.5.2 Demand

The demand for this type of intern mainly focuses on ensuring the accuracy of AI, providing risk warnings, protecting privacy, and clearly defining usage boundaries, to enhance their sense of security regarding AI-assisted tools and reduce concerns about using them due to fears of incorrect content, judgment errors, or information leakage.

S06: “I do not fully trust what AI-assisted tools tell me, especially when it’s about assessing a patient’s condition or deciding on care. I still check with my teacher first, because I’m afraid I might get it wrong and put the patient at risk.”

S13: “For simple reminders or looking things up, I think AI-assisted tools are fine as a reference. But when it comes to nursing diagnosis, risk assessment, or anything to do with patient safety, I’m very careful and will not rely on them alone.”

S24: “I am particularly concerned about patient information security. Especially when inputting patient data into some AI-assisted tools, I am worried about privacy leakage. Therefore, I will be particularly cautious about this type of content.”

## 4 Discussion

### 4.1 Differences in clinical nursing interns’ perceptions of using AI-assisted tools

This study found that the differences in the perception of AI-assisted tools by clinical nursing interns are mainly closely related to AI literacy, the maturity of clinical thinking, and the ability to apply contextualized responsibilities in clinical settings. In this study, The AI literacy maps closely onto the competencies set out in digital and AI literacy frameworks (12, 13). The maturity of clinical thinking shapes whether interns find AI genuinely useful and appraise it critically, which lies at the heart of technology acceptance (11). The ability to apply contextual responsibility, in turn, draws on the reliability and accountability that anchor trust in AI (14, 15). Firstly, AI literacy forms the basis for developing technical cognition. A recent multicenter study reported a similar pattern: healthcare and nursing students varied widely in their knowledge, perceptions, attitudes, and AI literacy, and thus in their readiness to use AI (10). Long and Magerko (19) stated that AI literacy is manifested as an individual’s ability to understand, use, evaluate, and critically reflect on AI. Interns with higher AI literacy are more capable of recognizing the auxiliary role of AI and making judgments and conversions on its output by combining the patient context, professional knowledge, and clinical requirements (20). Secondly, the maturity of clinical thinking affects whether interns can convert AI information into clinical learning outcomes (21, 22). This is consistent with recent reviews of ChatGPT and generative AI in nursing education, which credit these tools with supporting personalized learning, knowledge organization, and simulation-based training (23). Their educational value, though, still depends on whether students can critically evaluate and contextualize AI-generated content. Those with more mature clinical thinking are more likely to focus on whether AI-assisted tools can help them understand disease mechanisms, sort out nursing issues, and improve health education. They will incorporate the information from AI-assisted tools into their understanding of the patient’s condition and their individualized care plans. Meanwhile, the application ability of clinical contextual responsibility will affect interns’ perception of AI-assisted tools. Those with stronger such ability can integrate AI-assisted tools into the processes of nursing records, assessments, and verifications, and balance efficiency, standardization, and safety boundaries during their use (24). Those who lack this ability are likely to view AI as an additional burden or an uncertain factor due to mismatches in the process, operational burdens, privacy concerns, or liability risks (25). Furthermore, clear support and guidance are crucial conditions for promoting interns to use AI-assisted tools in a standardized manner. In a human–AI collaboration model, AI-assisted tools work best as supports within nursing workflows, backed by clear role boundaries, human oversight, and professional judgment (26). They should complement interns’ clinical reasoning, not replace it. Therefore, in the future, teaching and management strategies should be optimized from four aspects: AI boundary cognition, nursing judgment cultivation, clinical contextualized standardized application, and continuous support and guidance. This will provide a reference for optimizing the nursing internship teaching model and enhancing the human–AI collaborative practice ability of clinical nursing interns.

### 4.2 Persona characteristics of clinical nursing interns’ perceptions of using AI-assisted tools

#### 4.2.1 Active-thinking type

Among the active-thinking interns, comparatively high digital health literacy went together with earlier, largely self-directed use of AI, longer clinical placements, and time in more complex, higher-acuity wards. The active-thinking interns have a more positive perception of AI-assisted tools, mainly due to their strong thinking ability, proactive learning attitude, reflective tendency and sense of responsibility boundaries. In this group, most interns were undergraduate students and had more than 6 months of internship experience, and some had internship experience in emergency or ICU departments. This group also reported higher digital confidence, with more interns saying they could use AI-assisted tools on their own. Their longer clinical exposure, stronger learning base, and ease with digital tools likely helped them turn AI information into clinical reasoning, instead of using AI outputs as the answer. These clinical settings require rapid information integration, timely judgment, and high awareness of patient safety, which may further support their active learning tendency, reflective thinking, and ability to transform tool-based information into nursing judgments. Their thinking ability and proactive learning attitude make them more inclined to use AI-assisted tools in nursing scenarios such as patient assessment, nursing problems, nursing records and health education. During the process of screening, comparing and integrating the tool-based information, they gradually understand the applicable scope, usage boundaries and potential risks of AI-assisted tools, thereby promoting the improvement of AI literacy. Previous studies (27) have indicated that active learning helps learners conduct autonomous exploration, knowledge integration, and meaning construction, and enhances their critical thinking skills. The reflective tendency further enables them to link information provided by AI-assisted tools with patient situations, nursing norms, and supervisory feedback, transforming AI-assisted nursing tools into auxiliary resources that promote the development of nursing judgment and clinical thinking. Meanwhile, the strong sense of responsibility of this type of interns is related to their role identification and clinical immersion (28). They can understand the use of AI-assisted tools from the perspective of the nursing role, recognize that tool-based information may affect patient safety and privacy, and therefore are more likely to develop a cautious usage awareness. Badawy et al. (29) conducted a study that confirmed this view. These interns weigh AI output against the patient in front of them instead of taking it at face value, which is just what AI-literacy work means by evaluating and appropriately using AI rather than only operating it (13). Their trust is conditional too: they grant it only after judging whether a tool is accurate, open about its workings, and being used within its limits, much as recent multidimensional models of AI trust would predict (15). Therefore, the demand for such clinical nursing interns should be strengthened through structured clinical reasoning training based on their active exploration of AI-assisted tools, guiding them to first make their own nursing judgments, and then compare these judgments with the information, prompts, risk alerts, assessment results, or care suggestions provided by AI-assisted tools, as well as nursing standards, patients’ actual conditions, and feedback from instructors, in order to enhance their clinical judgment and application of norms under human–AI collaboration.

#### 4.2.2 Process-adaptation type

Process-adaptation interns came to AI mostly through what the ward built into the daily routine, and their digital skills stayed at a working level that still needed guidance. Process-adaptation interns have a more standardized understanding of AI-assisted tools, mainly due to their adaptation needs during the clinical transition phase, their rule awareness, and external support and guidance. In this group, their frequency of AI-assisted tool use was mainly concentrated at 3–5 times per week, suggesting that their perception of AI-assisted tools may be shaped by continuous exposure to routine clinical workflows and departmental requirements. Most interns in this group could use common digital tools when shown how, and several had received bedside demonstrations or departmental instruction on AI-assisted tools. This points to how their persona formed: through repeated workflow exposure and hands-on guidance from instructors, the tools came to feel like part of standardized clinical practice instead of something to explore on their own. After entering the clinical setting, interns need to shift from the school’s learning environment to the clinical field, facing new work rhythms, job requirements, and departmental norms. This often leads to confusion. Therefore, following the requirements can to some extent reduce the uncertainty in practice. Previous studies have indicated (30, 31) that a clear clinical learning environment and effective organizational support can help nursing students complete their role adaptation. At the same time, clear support guidance is an important condition for such interns to develop the ability to apply norms. Since its understanding of AI-assisted tools mainly relies on clinical procedures and teaching requirements, clear usage scenarios, verification standards, and responsibility boundaries can help incorporate the use of AI-assisted tools into clinical norms. For this group, what mattered was less personal motivation than the pull of the setting: departmental requirements and instructor demonstrations made the tools feel both expected and easy to use, the social influence and facilitating conditions that extended technology-acceptance models treat as key drivers of uptake (11). Thei reliance on this guided, step-by-step use also places their digital competence at the basic operational level described in frameworks such as DigComp 3.0 (12). Therefore, for process-adaptive interns, more process-integrated support and guidance should be provided. Internship departments can transform AI-assisted tool use into clear operational routines by providing standardized operation paths, checklist-based instructions, and scenario-specific demonstrations for common tasks such as nursing documentation, bedside information verification, risk assessment, and system reminders. The use boundaries, verification requirements, and responsibility norms of AI-assisted tools should be clarified within these routine workflows, helping them develop contextualized application capabilities in adapting to the clinical process.

#### 4.2.3 Task-dependent type

Among the task-dependent interns, heavy workloads and documentation pressure were accompanied by frequent AI use and a limited ability to evaluate its output. Task-dependent interns mainly perceive AI-assisted tools as tools for task completion and efficiency improvement. Their dependence is mainly related to task pressure, insufficient understanding of AI boundaries, and weak integration of clinical thinking. In this group, interns used AI-assisted tools relatively frequently, with most using them more than five times per week. Several interns in this group also used ChatGPT or other generative AI tools heavily for learning and documentation. That kind of frequent use under task pressure likely deepened their reliance on AI-generated frameworks and templates. This suggests that task-dependent interns had developed a high-frequency and task-oriented pattern of AI use in clinical practice, which may be related to documentation requirements, case data organization, and time pressure during internship tasks. During clinical internships, they encounter numerous learning and practical requirements, which leads them to prioritize the role of AI-assisted tools in enhancing efficiency and quickly obtaining information. Due to their insufficient AI literacy, these interns have limited understanding of the applicable scope, accuracy, and potential risks of the output content from AI-assisted tools, making it difficult for them to effectively judge and screen the results. Passi and Vorvoreanu (32) pointed out in their research that a lack of AI literacy may affect users’ ability to evaluate AI results and increase their excessive trust in the technical outputs. Consistent with this concern, this study further found that task pressure, documentation requirements, and case data organization may reinforce interns’ reliance on AI-generated frameworks and standardized expressions. Furthermore, as its clinical thinking is still in the developmental stage and lacks a stable framework for integrating patient information, nursing knowledge and practical situations for judgment, the structured information provided by AI-assisted tools can easily be directly used to complete tasks, without being further transformed into clinical thinking training resources. The difficulty here is that usefulness outpaces literacy: these interns clearly perceive AI as a fast way to get tasks done (11), yet they lack the evaluating and critical-use skills that AI-literacy frameworks treat as essential (12), so their trust stays functional and is rarely tested against the patient’s actual condition. Therefore, efforts should be made to strengthen task-oriented rules for using AI-assisted tools and verification requirements, guiding task-dependent interns to use AI-assisted tools primarily for information organization, framework drafting, or standardized expression. Before adopting AI-assisted content, interns should be required to supplement patient-specific information and check whether the content is consistent with the patient’s condition, treatment plan, and nursing norms, promoting their shift from relying on results to improving task quality and nursing judgment ability through tool-assisted verification.

#### 4.2.4 Passive-burden type

The passive-burden interns combined limited digital literacy and confidence and little or no prior AI training with tools that fit poorly into everyday ward routines. Passive-burden type interns have a relatively negative perception of AI-assisted tools, mainly due to insufficient AI literacy, high clinical adaptation pressure, and weak ability to apply contextual responsibilities. In this group, no interns reported high-frequency use of AI-assisted tools, which is consistent with their relatively low initiative in use and their tendency to use AI mainly in response to necessary workflow requirements. Most interns in this group had no specific AI training and felt unsure using digital or AI-assisted tools. That gap likely shaped how they saw the tools, as an extra operational burden rather than a help. When dealing with clinical process requirements and information technology operations, these interns tend to view AI-assisted tools as additional tasks. At the same time, because they have insufficient understanding of the function positioning, usage boundaries, and risk prevention of AI-assisted tools, they find it difficult to judge the reliability and applicability of the tool’s output, thereby generating uncertainty and a sense of exclusion. Dallora et al. (33) pointed out that insufficient understanding of technology and inadequate digital skills would reduce users’ acceptance of technology and increase their anxiety about using it. Consistent with previous findings, this study further indicated that passive-burden interns’ negative perceptions were also related to repeated operations, system reminders, and additional documentation burden in clinical workflows. Therefore, such interns often experience anxiety. At the same time, the clinical thinking and responsibility application abilities of these interns have not been fully developed, making it difficult for them to combine AI-assisted tools with patient situations, nursing norms, and supervisory feedback. They also fail to recognize the supportive value of AI-assisted tools for nursing judgment and learning processes. When digital and AI skills are thin, as they were in this group, the same tools that help others feel hard to operate, and low perceived ease of use drags acceptance down while feeding anxiety (11). With competence near the entry level of digital-competence frameworks (12), the absence of AI training and the gap between system design and ward workflow turn the tools into one more thing to manage rather than a support. Therefore, for passive-burden type interns, it is necessary to focus on redesigning the way AI-assisted tools are introduced and used in clinical workflows to help them correctly understand the supporting role and usage boundaries of AI-assisted tools, reduce resistance, and gradually form a positive and standardized application awareness. Clinical departments should establish graded use requirements according to interns’ clinical adaptation stage, allowing them to first master basic workflow-related functions and then gradually use more complex assessment or decision-support functions under supervision.

#### 4.2.5 Doubtful-defense type

Across different backgrounds, the doubtful-defense interns shared an absence of formal AI training and a strong sense of clinical responsibility. The cautious cognitive approach of the doubtful-defense type interns towards AI-assisted tools is mainly attributed to unclear understanding of AI boundaries, strong clinical responsibility awareness but insufficient contextual transformation capabilities. These interns usually recognize that AI-assisted tools may involve issues such as information accuracy, privacy security, responsibility attribution, and patient risks. None of the interns in this group had received specific AI training. Without that structured grounding, they were left more uncertain about how reliable AI-assisted tools are, where the tools apply, and who is accountable for their use. However, they lack a clear judgment on the source, applicable conditions, and limitations of the content generated by AI-assisted tools, thus finding it difficult to establish stable technical trust. This is consistent with the research results of Freyer et al. (34). In addition, these types of interns have a certain sense of clinical responsibility, but their maturity of clinical thinking and their ability to apply contextual responsibility still need to be further improved. When they have not yet formed a relatively stable nursing judgment framework, they are prone to interpret AI risks as overall unreliability and thus adopt an evasive or defensive attitude; when there are no clear verification standards and application paths, it is also difficult for them to transform their risk awareness into standardized usage behavior. Benzinger et al. (35) also found in their research that the application of medical AI needs to be carried out under the framework of human supervision, professional judgment, and ethical responsibility. For these interns the barrier is trust rather than skill. Their wariness centers on specific worries about accuracy, transparency, and who is accountable when AI is wrong, the dimensions that multidimensional accounts of AI trust regard as central (14, 15), rather than on any blanket rejection of the technology. Having had no formal AI training, they had no clear standards for verifying output, so a reasonable caution hardened into broad avoidance. Therefore, for doubtful defensive interns, it is necessary to strengthen the training on clarifying the applicable boundaries, verification pathways, and responsibility attribution of AI-assisted tools. This should guide them to verify the information, prompts, risk alerts, assessment results, or care suggestions provided by AI-assisted tools based on patient situations, nursing standards, and feedback from mentors, helping them convert risk sensitivity into prudent and standardized human–AI collaboration behaviors.

**Figure 3:**
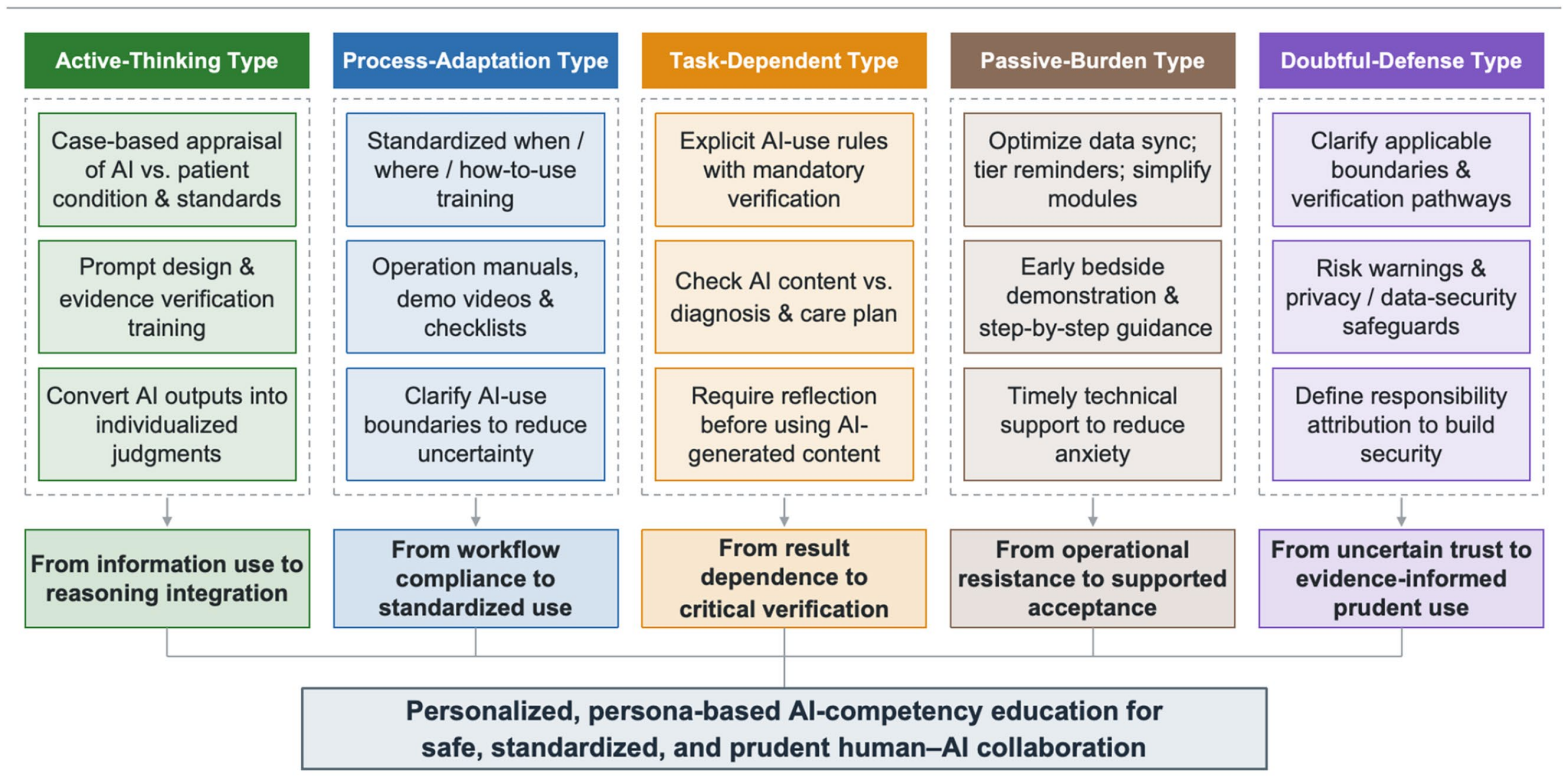
Persona-based educational interventions for human–AI collaboration.

Drawing on the support needs of the five persona types, we built a persona-based framework of educational interventions for human–AI collaboration (Figure 3). It pairs each persona with targeted teaching strategies and the learning shifts they are expected to make.

### 4.3 Ethical considerations in AI-assisted nursing education

AI-assisted tools raise several ethical concerns in nursing education. Hardie et al. (36) tie the use of generative AI in this setting to privacy, transparency, responsibility, and safe educational integration. Privacy is the most immediate. Identifiable patient data should never be entered into open or unsecured AI systems, and institutions need clear rules on who can access such data and how. Accuracy is a second concern. Fareed et al. (37) similarly caution that large language models in healthcare can return unreliable outputs, which is why their results need human oversight and verification before any clinical or educational use. So AI content must be checked against the patient’s condition, nursing standards, and instructor feedback. Accountability matters too. These tools can inform a decision but cannot replace professional judgment or supervised clinical responsibility. Tung et al. (38) add data privacy risks, unresolved legal liability, and algorithmic bias as further challenges in healthcare uses of generative AI. AI-related nursing education should build in all of these: privacy protection, output verification, bias awareness, clear responsibility boundaries, and safe human–AI collaboration.

### 4.4 Limitations

This study has certain limitations. Firstly, the research subjects were selected from three tertiary hospitals in Zhejiang and Jiangsu provinces, which did not cover more regions and different levels of hospitals. The generalizability of the research results still needs further verification. Future studies should include participants from diverse healthcare systems and cultural contexts. Secondly, the final sample size of this study was 25 nursing interns, although it reached data saturation, the sample size was still limited. In the future, the sample sources can be expanded. Thirdly, this study adopted a descriptive research method, and there may be certain subjective biases in the data analysis and character profiling process. Self-reporting bias, social desirability bias, and researcher interpretation bias may have influenced the findings. In the future, a multi-center, large-sample mixed study can be conducted to form a more accurate and stable character profile. Furthermore, future studies can develop and validate a questionnaire based on the persona dimensions identified in this study to quantitatively assess the distribution and influencing factors of different persona types among broader nursing populations.

## 5 Conclusion

This study identified five personas of clinical nursing interns in human–AI collaboration: active-thinking, process-adaptation, task-dependent, passive-burden, and doubtful-defense. These personas are useful mainly for teaching. Because interns differ in motivation, trust, dependence, risk awareness, and the help they need, AI training should fit the intern’s type instead of following one template. A persona-based approach lets nursing educators tailor that training, offering clinical reasoning support to some, workflow guidance or verification skills to others, and hands-on coaching or ethical risk education where needed. Applied this way, persona-based strategies can help interns use AI-assisted tools critically, safely, and responsibly, and support safe, standardized human–AI collaboration in nursing internship education.

## Data Availability

The original contributions presented in the study are included in the article/supplementary material, further inquiries can be directed to the corresponding authors.
